# Sugar Containing Compounds and Biological Activities of *Lagochilus setulosus*

**DOI:** 10.3390/molecules26061755

**Published:** 2021-03-21

**Authors:** Davlat Kh. Akramov, Nilufar Z. Mamadalieva, Andrea Porzel, Hidayat Hussain, Mthandazo Dube, Akbar Akhmedov, Ahmed E. Altyar, Mohamed L. Ashour, Ludger A. Wessjohann

**Affiliations:** 1 Institute of the Chemistry of Plant Substances, Uzbekistan Academy of Sciences, M. Ulugbek Str 77, Tashkent 100170, Uzbekistan; a.davlat@inbox.ru; 2 Department of Bioorganic Chemistry, Leibniz Institute of Plant Biochemistry, Weinberg 3, 06120 Halle, Germany; andrea.porzel@ipb-halle.de (A.P.); hidayat.hussain@ipb-halle.de (H.H.); mthandazo.dube@ipb-halle.de (M.D.); ludger.wessjohann@ipb-halle.de (L.A.W.); 3 Department of Botany and Plant Physiology, Samarkand State University, University Blv. 15, Samarkand 140104, Uzbekistan; rakbar@rambler.ru; 4 Department of Pharmacy Practice, Faculty of Pharmacy, King Abdulaziz University, Jeddah 21589, Saudi Arabia; aealtyar@kau.edu.sa; 5 Department of Pharmaceutical Sciences, Pharmacy Program, Batterjee Medical College, Jeddah 21442, Saudi Arabia; 6 Department of Pharmacognosy, Faculty of Pharmacy, Ain Shams University, Cairo 11566, Egypt

**Keywords:** Lamiaceae, *Lagochilus setulosus*, 1-Methoxy-3-*O*-*β*-glucopyranosyl-*α*-l-oliose, NMR, HR-MS, anthelmintic, antifungal, cytotoxic

## Abstract

Phytochemical investigation of the methanolic extract obtained from the aerial parts of *Lagochilus setulosus* (Lamiaceae) afforded the new compound 1-methoxy-3-*O*-*β*-glucopyranosyl-α-l-oliose (**1**) together with five known glycosides, namely sitosterol-3-*O*-*β*-glucoside (**2**), stigmasterol-3-*O*-*β*-glucoside (**3**), pinitol (**4**), 6*β*-hydroxyl-7-*epi*-loganin (**5**), and chlorotuberoside (**6**). The structures of these compounds were elucidated by extensive spectroscopic analyses, especially HR-MS, 1D and 2D NMR spectroscopy. The in vitro cytotoxic activity of the methanolic extract and the isolated compounds was assessed using 3-(4,5-dimethylthiazol-2-yl)-2,5-diphenyl tetrazolium bromide (MTT) and crystal violet (CV) staining assays. In addition, the antifungal activities of the components were evaluated against *Botrytis cinerea, Septoria tritici,* and *Phytophthora infestans.* The anthelmintic potential was determined against *Caenorhabditis elegans* nematodes. Neither the extract nor the isolated compounds showed promising activity in all the bioassays.

## 1. Introduction

The genus *Lagochilus* (Lamiaceae) is native to central, south-central, and eastern Asia. It consists of 46 species with 18 of them growing in Uzbekistan. Taxa of the genus *Lagochilus* basically occur throughout the territory of Uzbekistan, starting from the deserts to Tian-Shan and Pamir-Alay mountain systems. The majority of species can be found in the Pamir-Alai Mountain, southwest of Tian-Shan and Turanian lowland. Species from the genus *Lagochilus* belong to the most vulnerable plant species from the Lamiaceae family [1], with a decoction of herb and roots of *Lagochilus* species used in folk medicine mainly as styptics, hemostatics, and tranquilizers, and also for skin conditions, stomach pain, hemorrhages, and inflammation. Previous phytochemical investigations on *Lagochilus* species revealed the presence of essential oils, flavonoids, polysaccharides, sterols, iridoids, terpenoids, and more frequently diterpenes, which are considered as chemical markers for this genus. About 150 metabolites have been reported from all previous classes in the genus [2,3,4].

**Figure 1:**
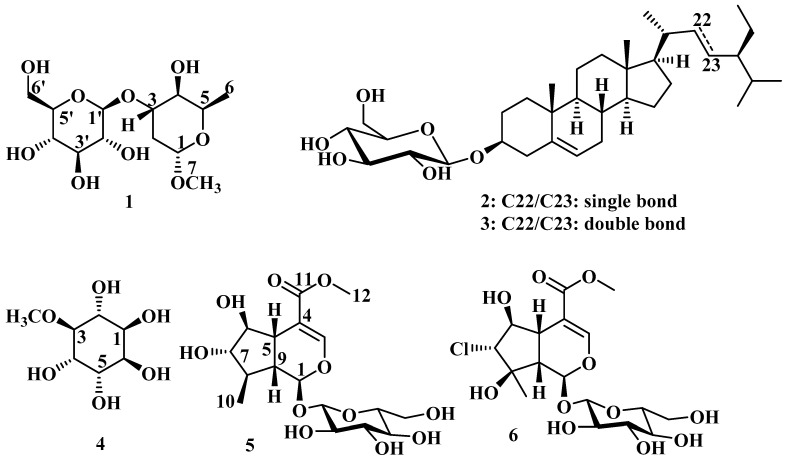
Chemical structures of the isolated compounds from the aerial parts of *L. setulosus.*

The species of *Lagochilus setulosus* Vved. is endemic of the southwestern area of Tian-Shan, and it can be found distributed in low mountains between the border of the regions Chimkent (Kazakhstan) and Tashkent (Uzbekistan) [1]. A literature survey reveals that there are no reports on traditional use, biological activity, or chemical investigations of *L. setulosus*, except our previous report on the volatile components of the aerial parts and their antioxidant and enzyme inhibitory activities [5]. In continuation of our phytochemical studies on *L. setulosus*, herein, we report the isolation and structure identification of a new disaccharide 1-methoxy-3-*O*-*β*-glucopyranosyl-α-l-oliose (**1**) (Figure 1) together with two known sterol glucosides: sitosterol-3-*O*-*β*-glucoside (daucosterol) (**2**) and stigmasterol-3-*O*-*β*-glucoside (**3**), as well as cyclitol–pinitol [3-(*O*-methyl-d-chiro-inositol)] (**4**), and the two iridoid glycosides 6*β*-hydroxyl-7-*epi*-loganin (**5**) and chlorotuberoside (**6**) (Figure 1). In addition, the anthelmintic, antifungal, and cytotoxic effects of the isolated compounds **1, 4,** a mixture of **5** and **6**, and the methanolic extract of *L. setulosus* have been evaluated.

## 2. Results and Discussion

### 2.1. Chemical Composition of L. setulosus

Since no publications were found regarding the chemical composition of the aerial parts of *L. setulosus*, this is the first report on the occurrence of sugar-containing compounds **1–6**. The chromatographic fractionation of the methanolic extract of this species afforded sitosterol-3-*O*-*β*-d-glucoside (**2**) and stigmasterol-3-*O*-*β*-d-glucoside (**3**) as an inseparable mixture (with a ratio of 1:1), and the new compound **1**. The chemical structure of compounds **2** and **3** was determined based on spectral analysis and comparisons with relevant literature [6]. These compounds were also found in other *Lagochilus* species viz. *L. inebrians* and *L. gypsaceus* [7]. Further investigations of the methanolic extract of *L. setulosus* afforded pinitol (**4**) and an inseparable mixture (with a ratio of 1.2:1) of 6*β*-hydroxyl-7-*epi*-loganin (**5**) and chlorotuberoside (**6**). Most recently, the iridoid glycoside **5** has been reported from several species of *Eremostachys* [8,9] and *Picconia excelsa* [10]. Chlorotuberoside (**6**) was previously isolated from different genera of Lamiaceae, such as *Phlomis* [11,12,13], *Eremostachys* [14], *Lamiophlomis* [15], which are taxonomically close to *Lagochilus*. Pinitol (**4**) is a cyclitol [16] and has been detected in gymnosperm (Pinus, Abies) and angiosperm (Fabaceae, Nyctaginaceae, Asteraceae, Zygophyllaceae, Caryophyllaceae, Aristolochiaceae, Sapindaceae, Santalaceae, and Aizoaceae) families [17]. Compounds **1**, **3**–**6** were isolated for the first time from the genus *Lagochilus*.

**Figure 2:**
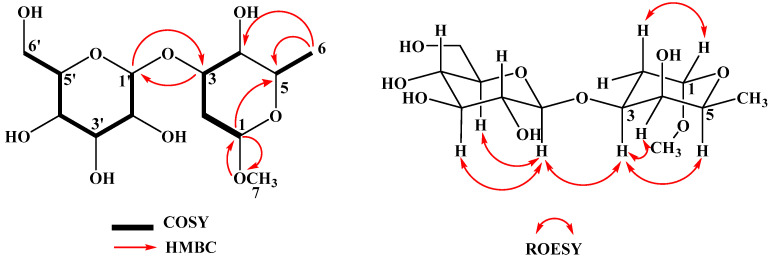
Selected 2D NMR correlations for 1-methoxy-3-*O*-*β*-glucopyranosyl-*α*-l-oliose (**1**).

Compound **1** was obtained as a white powder soluble in methanol. Its molecular formula C_13_H_24_O_9_ was deduced from its negative mode HR-ESI-MS showing the deprotonated molecular ion peak [M-H]^−^ at *m*/*z* 323.1336 (calcd. 323.1348, for C_13_H_23_O_9_^−^) (Supplementary Figures S1 and S2). The ^1^H and HSQC spectra of **1** show typical signals of two anomeric centres (*δ*H/*δ*C 4.77/100.1 ppm; *δ*H/*δ*C 4.38/102.8 ppm), a CH_3_ signal (*δ*H/*δ*C 1.23/17.1 ppm), and a methoxy signal (*δ*H/*δ*C 3.30/55.0 ppm). Together with the molecular formula C_13_H_24_O_9_ suggests the presence of a disaccharide including one deoxy sugar. COSY correlations and vicinal ^1^H-^1^H coupling constants indicate the presence of a *β*-d-glucose unit (Díaz et al. 2019). For the second sugar moiety, two spin systems were found (H-1/H-2A/B-H3-H4 and H-5/CH_3_-6) (Figure 2). HMBC correlations of the methyl group protons with both carbon signals C-5 (67.2 ppm) and C-4 (71.4 ppm), as well as the correlation of the methoxy protons with C-1 (100.1 ppm), revealed the presence of a 1-methoxy-2,6-dideoxy sugar unit. Vicinal ^1^H,^1^H coupling constants *δ*H 4.38 (1H, d, *J* = 7.7 Hz, H-1′) and *δ*H 4.09 (1H, ddd, *J* = 11.4, 7.7, 2.9 Hz) (Supplementary Table S1), and ROESY correlations between H-3/H-4 and H-3/H-5 show OR-3, OR-4 and CH_3_-6 positioned at one face and OMe-7 on the other face of the molecule. 2,6-Dideoxy glycosides and their methyl ethers occur as glycosides in natural products and the majority of them contains 1→3 or 1→4 linkages [18,19]. HMBC correlations of H-3/C-1′, as well as H-1′/C-3, confirmed compound **1** as 1-methoxy-3-*O*-*β*-glucopyranosyl-*α*-l-oliose (or 1-methoxy-3-*O*-*β*-d-glucopyranosyl-2,6-dideoxy-*α*-lyxo-hexopyranose). Detailed NMR spectra of compound **1** are included in the Supplementary data (Supplementary Figures S3–S8).

### 2.2. Biological Activities

The cytotoxicity of the crude methanolic extract of *L. setulosus*, compounds **1, 4**, and the mixture of **5** and **6** was evaluated in MTT and CV assays against the human cancer cell lines PC-3 (prostate cancer) and HT-29 (colon adenocarcinoma). Results of the tests showed weak effects. In addition, the methanolic extract and isolated compounds were tested against the phytopathogenic ascomycetes *Botrytis cinerea* Pers. and *Septoria tritici* Desm. and the oomycete *Phytophthora infestans* (Mont.) de Bary., but the samples did not show any promising activity up to the highest tested concentration of 125 ppm. The results of anthelmintic assays showed that the samples did not significantly kill *Caenorhabditis elegans* under the testing concentration of 500 μg/mL.

## 3. Materials and Methods

### 3.1. General Methods

UV-visible spectra of samples diluted in MeOH were obtained using a Jasco V770 UV/Vis spectrophotometer (JASCO GmbH, Pfungstadt, Germany). IR spectra were recorded with a Thermo Nicolet 5700 FT-IR spectrometer (Thermo Electron Corporation, Langenselbold, Germany). The HR-MS measurements were performed on a SciexTripleTOF 6600 LC-MS spectrometer (AB Sciex, Darmstadt, Germany) with an Acquity UPLC System (Waters GmbH, Eschborn, Germany) equipped with EC 150/2 NUCLEOSHELL RP18 column (150 × 2 mm, particle size 2.7 µm). The samples were measured in the negative and positive ion modes. 1D and 2D NMR spectra were recorded on a Varian/Agilent 400 and 600 NMR spectrometer (Agilent Technologies, Santa Clara, CA, USA). CD_3_OD and C_5_D_5_N were used as NMR solvent. Chemical shifts (*δ*) are referenced to internal tetramethylsilane (TMS) (*δ* = 0 ppm, ^1^H) and internal CD_3_OD (*δ* = 49.0 ppm, ^13^C) or internal pyridine-d5 (*δ* = 123.5 ppm, ^13^C). Thin-layer chromatography was performed on Merck pre-coated silica gel 60 F_254_ aluminum foil plates (Merck, Darmstadt, Germany). Spots were detected on TLC under UV lamp (254 and 365 nm) or by heating to 100 °C after spraying with vanillin sulfuric solution. Eluting solvents (MeOH, CHCl_3_, EtOAc) were distilled before use.

### 3.2. Plant Material

The aerial parts (flowers, leaves, and stems) of *L. setulosus* were collected in Oqtepa Yunusabad, Tashkent region (Uzbekistan), in May 2019. A voucher specimen (N273) is deposited at the Herbarium the Botany Institute of Uzbekistan and verified by Dr. Akbar Akhmedov. The aerial parts were air-dried in shadow and powdered in a mortar before use (moisture content was 11%, *w*/*w*).

### 3.3. Extraction and Isolation

Powdered aerial parts of *L. setulosus* (380 g) were extracted with MeOH (4 × 1.5 L). The resulting methanolic extracts were combined and evaporated at 40 °C to afford dry extract. The crude methanolic extract (41 g) was mixed with silica gel and placed onto a chromatography column (CC) (column size 10 × 80 cm) of silica gel (100–200 mesh, Tianjin Sinomed Pharmaceutical, China). The extract was eluted by CHCl_3_/MeOH gradients with increasing polarity to 20% MeOH to give 3 fractions A (100% CHCl_3_, 800 mL), B (CHCl_3_:MeOH, 9:1, *v*/*v*, 1.2 L) and C (CHCl_3_:MeOH, 4:1, *v*/*v*, 1.2 L). Fraction B (11.7 g) was re-chromatographed on silica gel CC (column size 6 × 65 cm) using CHCl_3_/MeOH in gradient mode of elution analysis and B1–13 subfractions (each 200 mL) were collected. Evaporation of the solvents from the subfractions, B3–4 and purification by PTLC produced a mixture of the compounds **2** and **3** (1.1 mg). The subfractions B10–11 (156 mg) were separated over silica gel using CHCl_3_:MeOH (9:1, *v*/*v*, 300 mL) to isolate **1**. Compound **1** (8 mg) was purified from the fraction using recrystallization in chloroform. Fr. C (8.8 g) was subjected to CC (silica gel, column size 4.2 × 70 cm) and eluted with EtOAc/MeOH gradient increasing the polarity to 20% MeOH. This yielded 23 subfractions which were combined into five groups (C1-C5). Subfr. C4 (540 mg) was subjected to CC (silica gel, column size 2 × 70 cm, EtOAc/MeOH: 10:1, *v*/*v*, (200 mL), 5:1, *v*/*v*, (200 mL) and 1:1, *v*/*v*, (700 mL) to obtain subfr. C4.1-C4.18. Subfr. C4.5-C4.7 gave mixture **5** and **6** (8 mg), while subfr. C4.15-C4.16 yielded pure **4** (12 mg).

### 3.4. Physical Properties of Isolated Compounds

1-Methoxy-3-*O*-β-*glucopyranosyl*-α-l-oliose (**1**), C_13_H_24_O_9_, Mr = 324 g/mol. White crystallin powder; UV λ_max_ (MeOH) nm: 263 nm. IR ν_max_ cm^−1^: 3356, 2905, 2361, 1647, 1443, 1359, 1035. HR-ESI-MS: *m*/*z* 323.1336 [M-H]^+^: (calcd for C_13_H_23_O_9_^+^, 323.1348); *m*/*z* 342.1784 [M + NH_4_]^+^: (calcd for C_13_H_28_NO_9_^+^, 342.1764); ^1^H NMR (CD_3_OD, 500 MHz) *δ*: 4.77 (1H, d, *J* = 2.3 Hz, H-1), 1.94 (1H, m, H-2a), 1.89 (1H, m, H-2b), 4.09 (1H, ddd, *J* = 11.4, 7.7, 2.9 Hz, H-3), 3.77 (1H, d, *J* = 2.8 Hz, H-4), 3.84 (1H, m, H-5), 1.23 (3H, d, *J* = 6.6 Hz, H-6), 3.30 (3H, s, H-7), 4.38 (1H, d, *J* = 7.7 Hz, H-1′), 3.19 (1H, dd, *J* = 8.9, 7.8 Hz, H-2′), 3.35 (1H, m, H-3′), 3.29 (1H, m, H-4′), 3.28 (1H, m, H-5′), 3.84 (1H, m, H-6′a), 3.68 (1H, dd, *J* = 11.9, 4.8 Hz, H-6′b). ^13^C NMR (CD_3_OD, 125 MHz) *δ*: 100.1 (C-1), 30.4 (C-2), 75.1 (C-3), 71.4 (C-4), 67.2 (C-5), 17.1 (C-6), 55.0 (C-7), 102.8 (C-1′), 75.0 (C-2′), 77.8 (C-3′), 71.4 (C-4′), 77.8 (C-5′), 62.5 (C-6′); UV, IR and NMR Spectra are available in the supplementary file.

Physical properties of the compounds **2–6** are given in the supplementary file.

### 3.5. Biological Assays

The anthelmintic, antifungal, and cytotoxic effects of all isolated compounds were determined by the procedures described below.

#### 3.5.1. Cytotoxic Activity

The cytotoxicity of the methanolic extract and isolated compounds was evaluated against the human tumor PC-3 (prostate cancer) and HT-29 (colon adenocarcinoma) cell lines. The extract and isolated compounds were tested at the concentrations of 0.05 and 50 μg/mL, and 0.01 and 10 μM, respectively. The cell maintenance and assay procedure were performed as described by Dos Santos et al. [20]. The viability of the cells was determined by MTT and CV assays after 72 h incubation time. The absorbance was measured with an automated microplate reader at 540 nm with a reference wavelength of 670 nm [21]. The results are presented as a percentage of control values obtained from untreated cultures.

#### 3.5.2. Antifungal Activity

The methanol extract and isolated compounds were tested in 96-well microtiter plate assays against the phytopathogenic ascomycetes *Botrytis cinerea* Pers. and *Septoria tritici* Desm. and the oomycete *Phytophthora infestans* (Mont.) de Bary according to the monitoring methods approved by the fungicide resistance action committee (FRAC) with minor modifications as described before [22]. Crude extracts and fractions were examined at a final concentration of 125 μg/mL, while pure compounds were tested in a serial dilution, ranging from 100 to 0.1 μM. The solvent DMSO was used as negative control (max. concentration 2.5%), and the commercially used fungicides epoxiconazole and terbinafine (Sigma-Aldrich, Darmstadt, Germany) served as positive controls. Five to seven days after inoculation, pathogen growth was evaluated by measurement of the optical density (OD) at *λ* 405 nm with a TecanGENios Pro microplate reader (5 measurements per well using multiple reads in a 3 × 3 square). Each experiment was carried out in triplicates.

#### 3.5.3. Anthelmintic Activity

The Bristol N2 wild type strain of *Caenorhabditis elegans* was used in the anthelmintic assay. The nematodes were cultured on NGM (Nematode Growth Media) petri plates using the uracil auxotroph *E. coli* strain OP50 as a food source according to the methods described before [20]. The anthelmintic assay was carried out following the method developed by Thomsen et al. [23]. Briefly, after 4 days of cultivation, the nematodes were transferred from the Petri plate to a 15 mL falcon tube by rinsing each plate twice with 2 mL M9 buffer. The worm suspension was centrifuged for 1 min at 800 G. After removal of the supernatant, the nematodes were washed again with 2 mL M9 buffer under the same conditions and, depending on the number of individuals, re-suspended in 2 to 8 mL M9 buffer. To this suspension, 10 μL penicillin-streptomycin-solution (10 mg/mL) was added. After triply counting the nematodes in 10 μL solution droplets under a stereo microscope (Olympus SZX12), the worm number was adjusted to 20–30 animals per 20 μL. The assay was performed in 384 well plates. The outer wells were filled with water to minimize evaporation. To the test wells, 20 μL worm suspension was added and the number of living and dead animals in each well were counted using the cell culture microscope Olympus CKX41. The number of living nematodes was set at 100%. At staggered intervals, 20 μL test solution (test compound in 4% DMSO in M9 buffer) was added followed by a microscopic enumeration of living and dead test organism after 30 min of incubation. For all test plates, the solvent DMSO (2%) and the standard anthelmintic drug ivermectin (10 μg/mL) were used as negative and positive controls, respectively. All the assays were done in triplicate.

## 4. Conclusions

From the aerial parts of *L. setulosus*, a new disaccharide, 1-methoxy-3-*O*-*β*-glucopyranosyl-*α*-l-oliose, and five known glycosides were isolated and identified. All compounds were isolated for the first time from this species. The results of anthelmintic, antifungal, and cytotoxic assays demonstrated that the methanolic extract and isolated compounds of *L. setulosus* were not toxic in general in in vitro assays.

## Data Availability

The data presented in this study are available in article and supplementary material.

## Supplementary Materials

The following are available online, Spectral data of the new compound **1** are available as Supporting Information (Tables S1–S4), (Figures S1–S10). Table S1: ^13^C and ^1^H NMR data of 1-methoxy-3-*O*-*β*-glucopyranosyl-*α*-l-oliose (**1**) (600 MHz, *δ*, ppm, in CD_3_OD), Table S2: ^13^C and ^1^H NMR data (*J* in Hz) of the compound **2** and **3** (400 MHz, *δ*, ppm, in C_5_D_5_N), Table S3: NMR spectroscopic data for 6*β*-hydroxyl-7-epi-loganin (**5**) (500 MHz, *δ*, ppm, *J*/Hz), Table S4: NMR spectroscopic data for chlorotuberoside (**6**) (500 MHz, *δ*, ppm, *J*/Hz). Figure S1: HR-ESI-QTOF-MS (+ve) spectrum of 1-methoxy-3-*O*-*β*-glucopyranosyl-*α*-l-oliose (**1**), Figure S2: HR-ESI-QTOF-MS (-ve) spectrum of 1-methoxy-3-*O*-*β*-glucopyranosyl-*α*-l-oliose (**1**), Figure S3: ^1^H NMR spectrum of 1-methoxy-3-*O*-*β*-glucopyranosyl-*α*-l-oliose (**1**) in CD_3_OD (o-oliose, g-glucose), Figure S4: ^13^C NMR spectrum of 1-methoxy-3-*O*-*β*-glucopyranosyl-*α*-l-oliose (**1**) in CD_3_OD, Figure S5: HSQC spectrum of 1-methoxy-3-*O*-*β*-glucopyranosyl-*α*-l-oliose (**1**) in CD_3_OD, Figure S6: HMBC spectrum of 1-methoxy-3-*O*-*β*-glucopyranosyl-*α*-l-oliose (**1**) in CD_3_OD, Figure S7: COSY spectrum of 1-methoxy-3-*O*-*β*-glucopyranosyl-*α*-l-oliose (**1**) in CD_3_OD, Figure S8: NOESY spectrum of 1-methoxy-3-*O*-*β*-glucopyranosyl-*α*-l-oliose (**1**) in CD_3_OD, Figure S9: IR spectrum of 1-methoxy-3-*O*-*β*-glucopyranosyl-*α*-l-oliose (**1**), Figure S10: UV spectrum of 1-methoxy-3-*O*-*β*-glucopyranosyl-*α*-l-oliose (**1**) in CH_3_OH.
