# Supplementary material for: Sugar Containing Compounds and Biological Activities of Lagochilus setulosus
Source: Molecules. 2021 Mar 21;26(6):1755. doi: 10.3390/molecules26061755 (PMC8004061; doi:10.3390/molecules26061755)
Supplement: Supplementary file 1 [file molecules-26-01755-s001.pdf]

Supplementary Material

Sugar containing compounds and biological activities of *Lagochilus setulosus*

Davlat Kh. Akramov [1], Nilufar Z. Mamadalieva [1,2,*], Andrea Porzel [2], Hidayat Hussain [2], Mthandazo Dube [2], Akbar Akhmedov [3], Ahmed E. Altyar [4], Mohamed L. Ashour [5,6,*], Ludger A. Wessjohann [2]

[1] Institute of the Chemistry of Plant Substances, Uzbekistan Academy of Sciences, M. Ulugbek Str 77, 100170 Tashkent, Uzbekistan; a.davlat@inbox.ru (D.Kh.A.), nmamadalieva@yahoo.com (N.Z.M.)

[2] Department of Bioorganic Chemistry, Leibniz Institute of Plant Biochemistry, Weinberg 3, 06120 Halle (Saale), Germany; andrea.porzel@ipb-halle.de (A.P.), hidayat.hussain@ipb-halle.de (H.H.), mthandazo.dube@ipb-halle.de (M.D.), ludger.wessjohann@ipb-halle.de (L.A.W.)

[3] Institute of Botany, Uzbekistan Academy of Sciences, Durmon Yuli Str 32, 100125 Tashkent, Uzbekistan; rakbar@rambler.ru (A.A.)

[4] Department of Pharmacy Practice, Faculty of Pharmacy, King Abdulaziz University, P.O. Box 80260 Jeddah- 21589, Saudi Arabia; aealtyar@kau.edu.sa

[5] Department of Pharmaceutical Sciences, Pharmacy Program, Batterjee Medical College, Jeddah 21442, Saudi Arabia; mohamed.ashour@bmc.edu.sa

[6] Department of Pharmacognosy, Faculty of Pharmacy, Ain Shams University, Cairo 11566, Egypt; ashour@pharma.asu.edu.eg

* Correspondence: nmamadalieva@yahoo.com (N.Z.M.), mohamed.ashour@bmc.edu.sa (M.L.A)

Additional Experimental Detail

Table S1: ^{13}C and ^{1}H NMR data of 1-methoxy-3-O-β-glucopyranosyl-α-L-oliose (**1**) (600 MHz, δ, ppm, in CD_3OD)

Table S2: ^{13}C and ^{1}H NMR data (J in Hz) of the compound **2** and **3** (400 MHz, δ ppm, in C_5D_5N)

Table S3: NMR spectroscopic data for 6β-hydroxyl-7-epi-loganin (**5**) (500 MHz, δ, ppm, J/Hz)

Table S4: NMR spectroscopic data for chlorotuberoside (**6**) (500 MHz, δ, ppm, J/Hz)

Figure S1: HR-ESI-QTOF-MS (+ve) spectrum of 1-methoxy-3-O-β-glucopyranosyl-α-L-oliose (**1**)

Figure S2: HR-ESI-QTOF-MS (-ve) spectrum of 1-methoxy-3-O-β-glucopyranosyl-α-L-oliose (**1**)

Figure S3: ^{1}H NMR spectrum of 1-methoxy-3-O-β-glucopyranosyl-α-L-oliose (**1**) in CD_3OD (o-oliose, g-glucose)

Figure S4: ^{13}C NMR spectrum of 1-methoxy-3-O-β-glucopyranosyl-α-L-oliose (**1**) in CD_3OD

Figure S5: HSQC spectrum of 1-methoxy-3-O-β-glucopyranosyl-α-L-oliose (**1**) in CD_3OD

Figure S6: HMBC spectrum of 1-methoxy-3-O-β-glucopyranosyl-α-L-oliose (**1**) in CD_3OD

Figure S7: COSY spectrum of 1-methoxy-3-O-β-glucopyranosyl-α-L-oliose (**1**) in CD_3OD

Figure S8: NOESY spectrum of 1-methoxy-3-O-β-glucopyranosyl-α-L-oliose (**1**) in CD_3OD

Figure S9: IR spectrum of 1-methoxy-3-O-β-glucopyranosyl-α-L-oliose (**1**) (spectra were measured in ATR mode)

Figure S10: UV spectrum of 1-methoxy-3-O-β-glucopyranosyl-α-L-oliose (**1**) in CH_3OH

3. Experimental

3.4. Physical properties of isolated compounds

1-Methoxy-3-O-β-glucopyranosyl-*α*-L-oliose (**1**), $C_{13}H_{24}O_9$, Mr = 324 g/mol. White crystallin powder; UV λ_{max} (MeOH) nm: 263 nm. IR ν_{max} cm^{-1}: 3356, 2905, 2361, 1647, 1443, 1359, 1035. HR-ESI-MS: *m/z* 323.1336 $[M-H]^+$: (calcd for $C_{13}H_{23}O_9^+$, 323.1348); *m/z* 342.1784 $[M+NH_4]^+$: (calcd for $C_{13}H_{28}NO_9^+$, 342.1764); ^{1}H and ^{13}C NMR chemical shifts see Table S1.

Table S1. ^{13}C and ^{1}H NMR data of 1-methoxy-3-O-β-glucopyranosyl-*α*-L-oliose (**1**) (600 MHz, δ, ppm, in CD_3OD)

C	APT	δC	δH	C	APT	δC	δH
Oliose				Glucose			
1	CH	100.1	4.77, d=2.3 Hz	1'	CH	102.8	4.38, d=7.7 Hz
2	CH_2	30.4	1.94 m, 1.87 m	2'	CH	75.0	3.19, dd=8.9, 7.7 Hz
3	CH	75.1	4.09, ddd=11.5, 5.7, 2.9 Hz	3'	CH	77.8	3.35, m
4	CH	71.4	3.77, d=2.8 Hz	4'	CH	71.4	3.29, m
5	CH	67.2	3.84, m	5'	CH	77.8	3.28, m
6	CH_3	17.1	1.23, d=6.6 Hz	6'	CH_2	62.5	3.84 m, 3.68, dd=11.9, 4.8 Hz
7	OCH_3	55.0	3.30, s				

Sitosterol-3-O-β-glucoside (Daucosterol) (2). $C_{35}H_{60}O_6$, Mr=576.44 g/mol, white powder. ^{1}H and ^{13}C NMR chemical shifts see Table S2.

Stigmasterol-3-O-β-glucoside (3). $C_{35}H_{58}O_6$, Mr=574.8 g/mol, white powder. ^{1}H and ^{13}C NMR chemical shifts see Table S2.

Table S2. ^{13}C and ^{1}H NMR data (J in Hz) of the compound **2** and **3** (400 MHz, δ ppm, in C_5D_5N)

	Sitosterol-3-O-β-glucoside (**2**)			Stigmasterol-3-O-β-glucoside (**3**)		
**C**	**APT**	**δC**	**δH**	**APT**	**δC**	**δH**
1	CH_2	37.48	1.74 (t, *J* = 11.8, 7.7 Hz), 1.00, m	CH_2	37.48	1.74 (t, *J* = 11.8, 7.7 Hz), 1.00, m
2	CH_2	29.46	1.76, m, 1.32, m	CH_2	29.46	1.76, m, 1.32, m
3	CH	78.10	3.97, m	CH	78.10	3.97, m
4	CH_2	39.35	2.75 (d, *J* = 13.0 Hz), 2.50 (t, *J* = 12.2 Hz)	CH_2	39.35	2.75 (d, *J* = 13.0 Hz), 2.50 (t, *J* = 12.2 Hz)
5	C	140.94		C	140.94	
6	CH	121.96	5.37 (d, *J* = 4.9 Hz)	CH	121.96	5.37 (d, *J* = 4.9 Hz)
7	CH_2	32.06	1.91, m, 1.54, m	CH_2	32.06	1.91, m, 1.54, m
8	CH	31.18	1.39, m	CH	31.18	1.39, m
9	CH	50.35	0.92, m	CH	50.35	0.92, m
10	C	36.39		C	36.39	
11	CH_2	21.30	1.45, m, 1.40, m	CH_2	21.30	1.45, m, 1.40, m
12	CH_2	39.82	1.99 (d, *J* = 12.8 Hz), 1.11, m	CH_2	39.82	1.99 (d, *J* = 12.8 Hz), 1.11, m
13	C	42.35		C	42.48	
14	CH	56.83	0.96, m	CH	56.92	0.96, m
15	CH_2	24.51	1.56, m, 1.04, m	CH_2	24.54	1.56, m, 1.04, m

16	CH_2	28.55	1.86, m, 1.27, m	CH_2	26.01	1.86, m, 1.27, m
17	CH	56.24	1.12, m	CH	56.06	1.12, m
18	CH_3	11.98	0.67, s	CH_3	12.14	0.69, s
19	CH_3	19.01	1.00, s	CH_3	19.08	
20	CH	36.93	1.41, m	CH	40.79	2.06
21	CH_3	19.21	0.95, (d, *J* = 6.4 Hz)	CH_3	21.29	0.92, d
22	CH_2	34.21	1.41, m, 1.09, m	CH	138.86	5.23 (dd, *J* = 15.2, 8.7 Hz)
23	CH_2	26.38	1.27 (2H)	CH	129.48	5.08 (d, *J* = 7.6 Hz)
24	CH	46.04	1.01	CH	51.42	1.60
25	CH	29.32	1.70, m	CH	31.18	
26	CH_3	19.18	0.88, (d, *J* = 6.8 Hz)	CH_3	19.99	0.95
27	CH_3	19.43	0.89, (d, *J* = 6.8 Hz)	CH_3	21.47	1.09
28	CH_2	23.39	1.30, m (2H)	CH_2	25.71	
29	CH_3	12.16	0.91, t	CH_3	12.53	0.99
Glucose						
	CH-1′	102.59	5.09 (d, J = 7.2 Hz)	CH-1′	102.59	5.09 (d, J = 7.2 Hz)
	CH-2′	75.37	4.09, d	CH-2′	75.37	4.09, d
	CH-3′	78.64	4.32, d	CH-3′	78.64	4.32, d
	CH-4′	71.71	4.31, d	CH-4′	71.71	4.31, d
	CH-5′	78.52	4.02, m	CH-5′	78.52	4.02, m
	CH2-6′	62.85	4.59 (d, J = 11.3 Hz), 4.44 (dd, J = 11.6, 5.8 Hz)	CH2-6′	62.85	4.59 (d, J = 11.3 Hz), 4.44 (dd, J = 11.6, 5.8 Hz)

Pinitol (4). $C_7H_{14}O_6$, Mr=194.18 g/mol, white powder. UV λ_{max} (MeOH): 271 nm. IR ν_{max} cm^{-1}: 3391, 3307, 2948, 2903, 1449, 1051, 1002, 749. HR-ESI-Q-TOF-MS: for $[M-H]^-$ found 193.0718, calc. 193.0712; for $[M+HCOO]^-$ found 239.0767, calc. 239.0767; for $[M+Na]^+$ found 217.0696, calc. 217.0688. ^{1}H NMR (400 MHz, pyridine-d5, δ, ppm, *J*/Hz): 4.93 (m, 1H, H-6), 4.80-5.00 (m, 3H, H-1, H-4, H-5), 4.66 (1H, td, *J* = 9.2, 3.1 Hz, H-2), 4.18 (1H, t, *J* = 9.2 Hz, H-3), 3.95 (3H, s, OMe). ^{13}C NMR (100 MHz, pyridine-d5, δ, ppm): 85.90 (C-3), 74.73 (C-1), 74.23 (C-4), 73.80 (C-6), 73.11 (C-5), 72.31 (C-2), 60.78 (OMe).

6β-Hydroxyl-7-epi-loganin (5). $C_{17}H_{26}O_{11}$, Mr=406.38 g/mol, white powder. HR-ESI-Q-TOF-MS: for $[M-H]^-$ found 405.1402, calc. 405.1397; for $[M+HCOO]^-$ found 451.1457, calc. 451.1452; for $[M+H]^+$ found 407.1548, calc. 407.1553. ^{1}H and ^{13}C NMR chemical shifts see Table S3.

Table S3. NMR spectroscopic data for 6β-hydroxy-7-epi-loganin (**5**) (500 MHz, δ, ppm, J/Hz)

C	APT	6β-Hydroxyl-7-epi-loganin (**5**) (CD_3OD)		6β-Hydroxyl-7-epi-loganin (**5**) (CD_3OD, 250 MHz, Damtoft et al., 1997)	
		**δH**	**δC**	**δH**	**δC**
1	CH	5.39, d, J=4.0 Hz	96.47	5.40, d, J=4.0 Hz	96.4
3	CH	7.43, d, J=1.4 Hz	153.02	7.44, d, J=1.2 Hz	153.0
4	C	-	111.23		111.2
5	CH	2.74, dd, J=1.3, 13.8 Hz	39.40	2.75, br dd, J=1.5, 9.0 Hz	39.4

6	CH	3.70	84.37	3.70	84.3
7	CH	3.46, dd, J=5.9, 8.8 Hz	85.81	3.46, dd, J=6.0, 8.5 Hz	85.8
8	CH	1.69, m	41.13	1.70, m	41.1
9	CH	2.03, dt, J=3.9, 3.9, 2.5 Hz	44.48	2.03, dt, J=4.0, 9.0 Hz	44.4
10	CH_3	1.15, s	17.19	1.16, d, J=6.5 Hz	17.2
11	CO	-	170.08		170.0
12	OCH_3	3.73, s	51.96	3.73, s	51.9
Glucose					
1'	CH	4.63, d=7.8 Hz	100.11	4.63, d, J=7.5 Hz	100.1
2'	CH	3.15, dd, J=7.6, 13.0 Hz	74.68	3.16, dd, J=8.0, 9.0 Hz	74.6
3'	CH	3.33, d, J= 3.0 Hz	78.01	3.40-3.25 (3H, H-3', H-4', H-5')	78.0
4'	CH	3.25, d, J=9.4 Hz	71.57		71.5
5'	CH	3.29, m	78.38		78.3
6'	CH_2	3.90, t, J=2.4 Hz<br>3.70, d, J=5.2 Hz	62.74	3.89, dd, J=2.0, 12.0 Hz<br>3.70	62.7

Chlorotuberoside (6). $C_{17}H_{25}ClO_{11}$, Mr=440.83 g/mol, white powder. HR-ESI-Q-TOF-MS: for $[M-H]^-$ found 439.1013, calc. 439.1007; for $[M+HCOO]^-$ found 485.1062, calc. 485.1067; for $[M+H]^+$ found 441.1158, calc. 441.1163. ^1H and ^{13}C NMR chemical shifts see Table S4.

Table S4. NMR spectroscopic data for chlorotuberoside (**6**) (500 MHz, δ, ppm, J/Hz)

C	APT	Chlorotuberoside (**6**) (CD_3OD)		Chlorotuberoside (**6**) (CD_3OD, 500 MHz, Calis et al., 2005)	
		**δH**	**δC**	**δH**	**δC**
1	CH	5.66, br s	93.34	5.66, br s	93.0
3	CH	7.41, d, J=1.3 Hz	152.33	7.41, d, J=1.0 Hz	152.0
4	C	-	111.72	-	111.3
5	CH	2.82, dd, J=4.3, 12.9 Hz	36.20	2.83, ddd, J=11.5, 4.3, 1.0 Hz	35.8
6	CH	3.68	82.48	3.67	82.1
7	CH	3.99, d, J=8.9 Hz	74.52	4.01, d, J=8.9 Hz	74.1
8	C	-	77.72	-	77.3
9	CH	2.66, d, J=11.4 Hz	47.89	2.66, d, J=11.5 Hz	47.5
10	CH_3	1.19, s	18.89	1.19, s	18.6
11	CO	-	169.46	-	169.1
12	OCH_3	3.74, s	52.00	3.74, s	51.6
Glucose					
1'	CH	4.62, d, J=8.3 Hz	99.77	4.61, d, J=7.9 Hz	99.3
2'	CH	3.17, dd, J=7.6, 9.2 Hz	74.56	3.14, dd, J=7.9, 8.9 Hz	74.1
3'	CH	3.35, dd, J=3.3, 8.6 Hz	77.95	3.35, t, J=8.9 Hz	77.5
4'	CH	3.29, m	71.55	3.28, t, J=8.9 Hz	71.7
5'	CH	3.29, m	78.33	3.30, m	77.9
6'	CH_2	3.88, d, J=2.4 Hz<br>3.64, d, J=5.8 Hz	62.74	3.88, dd, J=12.0, 2.0 Hz<br>3.66, dd, J=12.0, 5.9 Hz	62.3

molecules

MDPI

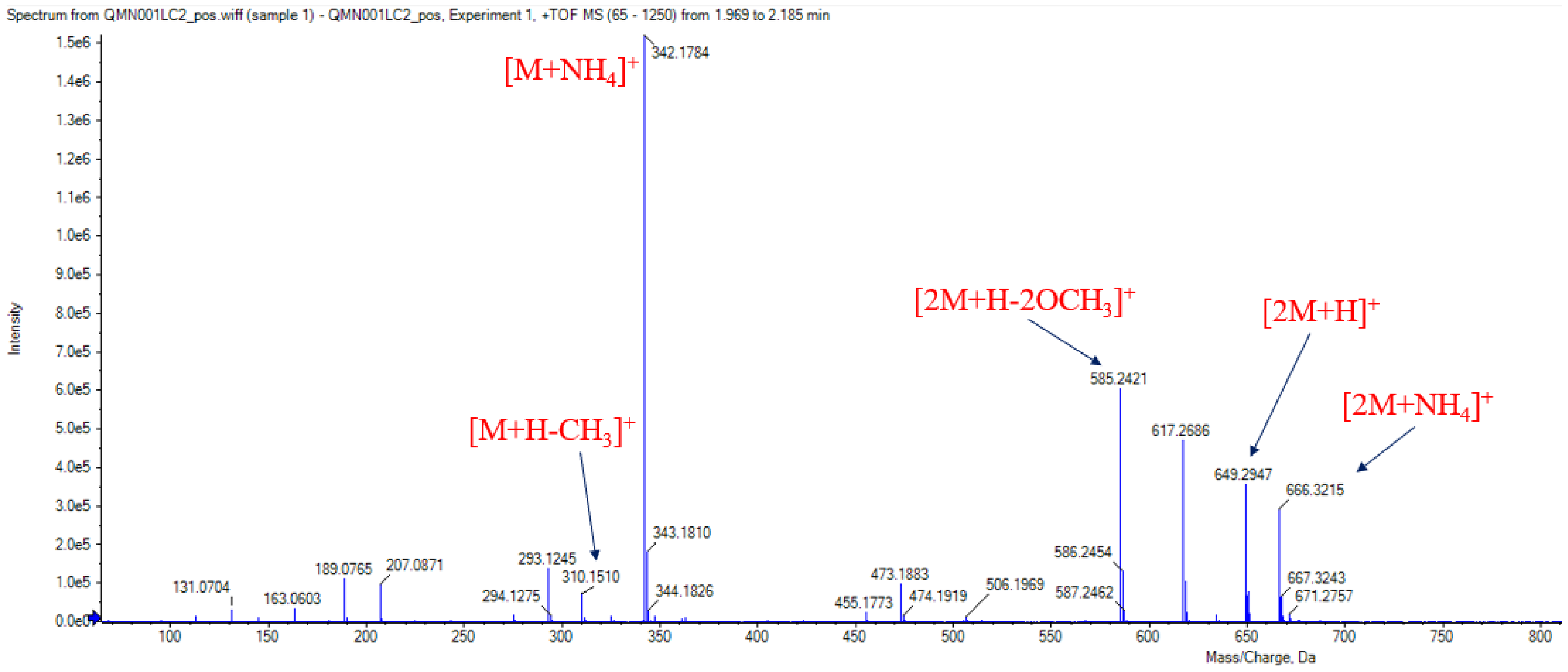

Figure S1. HR-ESI-QTOF-MS (+ve) spectrum of 1-methoxy-3-O-β-glucopyranosyl-α-L-oliose (**1**)

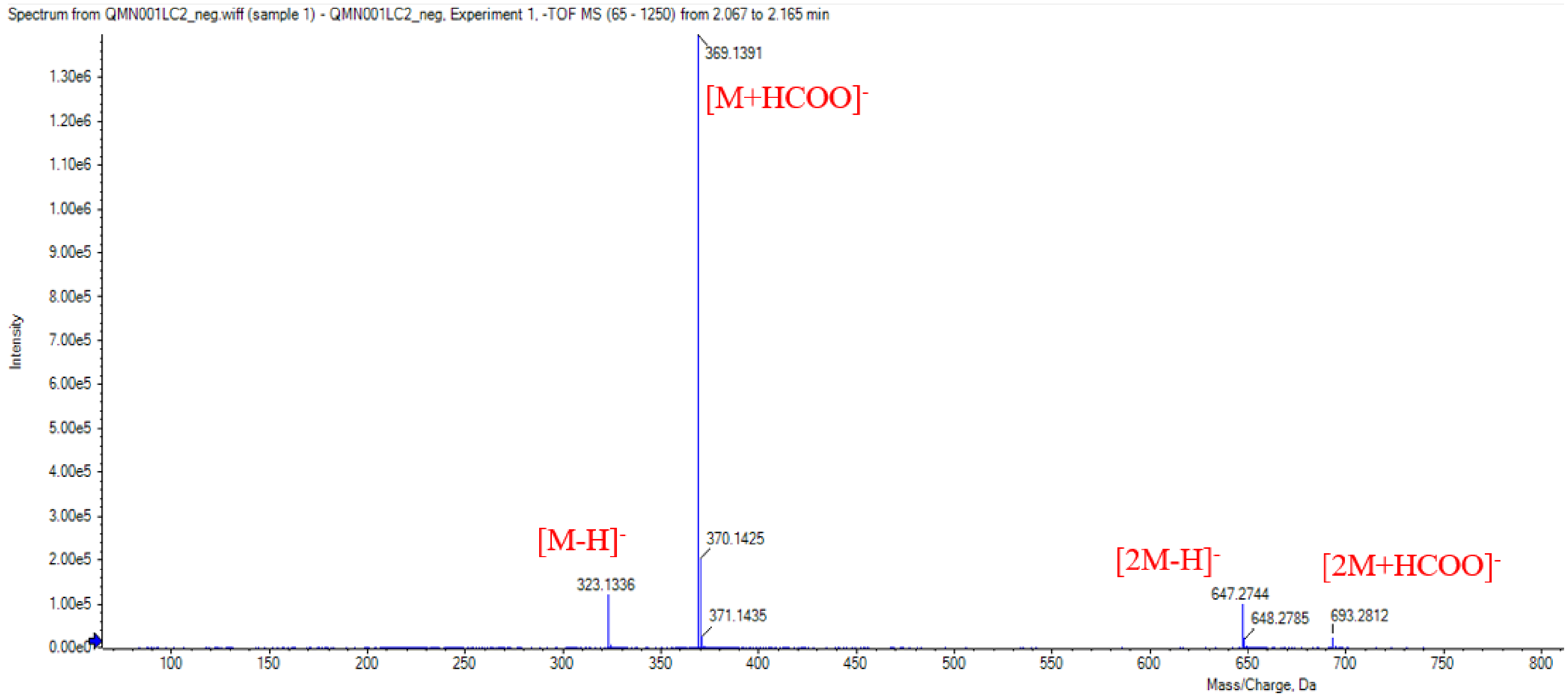

Figure S2. HR-ESI-QTOF-MS (-ve) spectrum of 1-methoxy-3-O-β-glucopyranosyl-α-L-oliose (**1**)

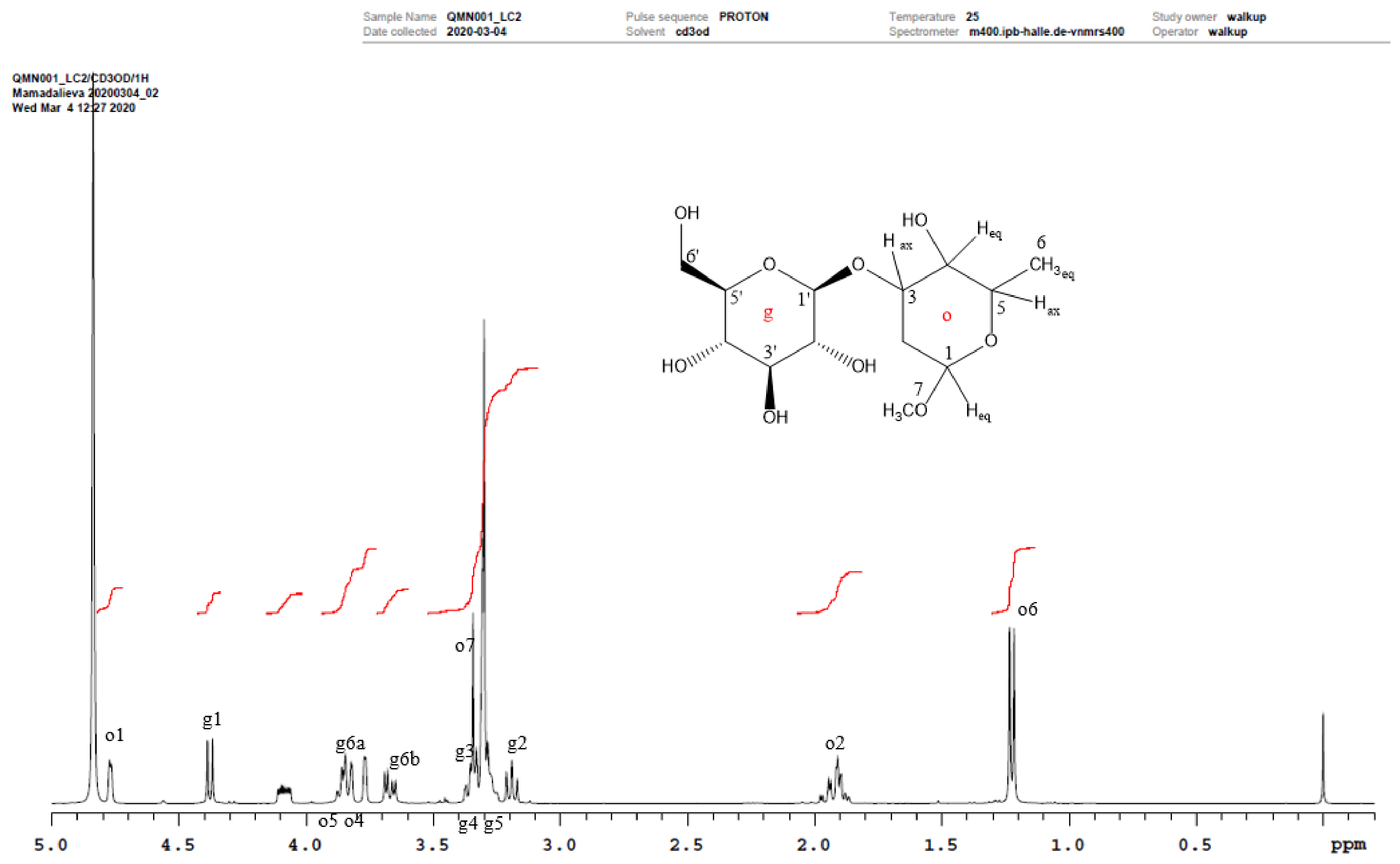

Figure S3. ^{1}H NMR spectrum of 1-methoxy-3-O-β-glucopyranosyl-α-L-oliose (**1**) in CD_3OD (o-oliose, g-glucose)

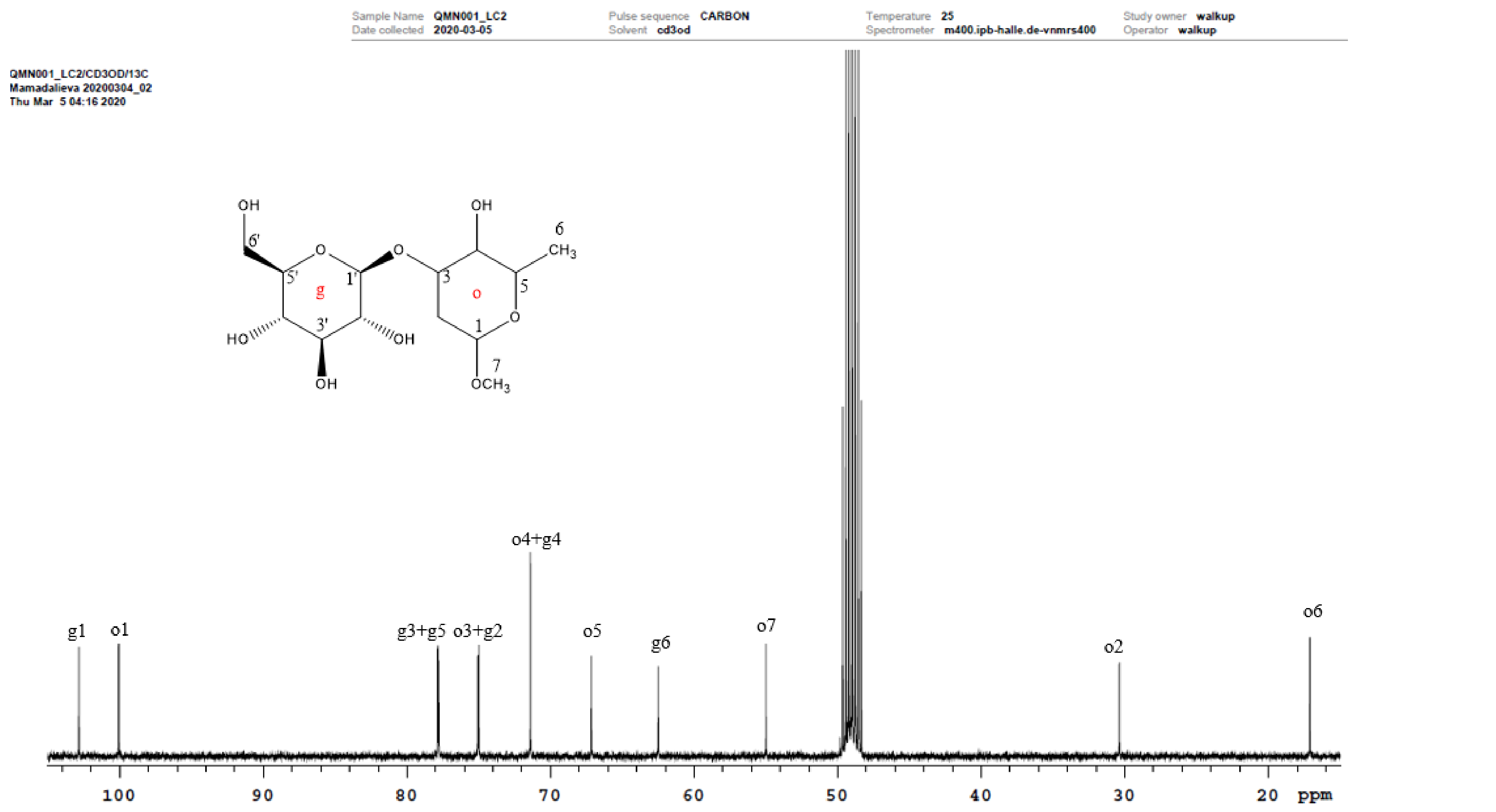

Figure S4. ^{13}C NMR spectrum of 1-methoxy-3-O-β-glucopyranosyl-α-L-oliose (**1**) in CD_3OD

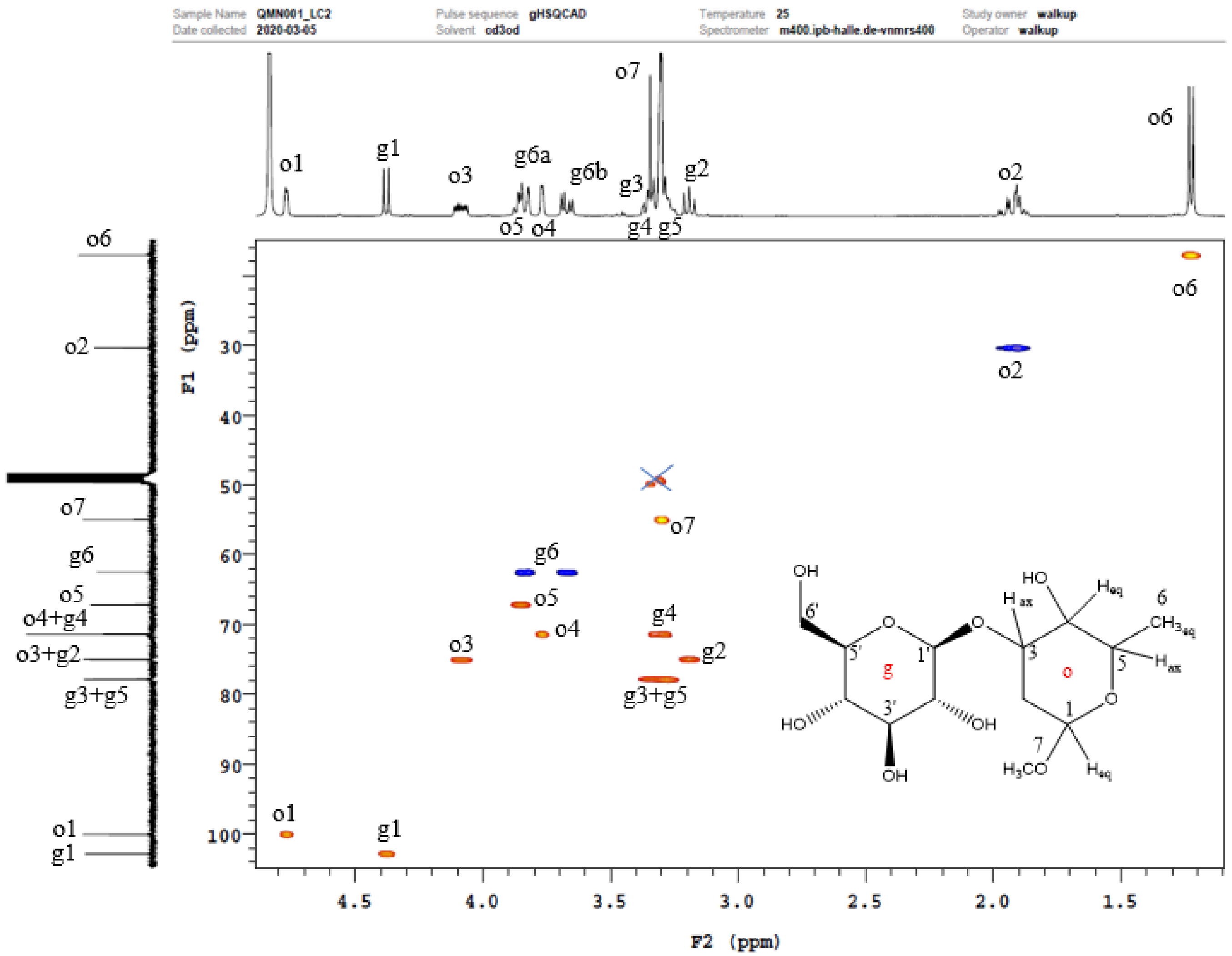

Figure S5. HSQC spectrum of 1-methoxy-3-O-β-glucopyranosyl-α-L-oliose (**1**) in CD3OD

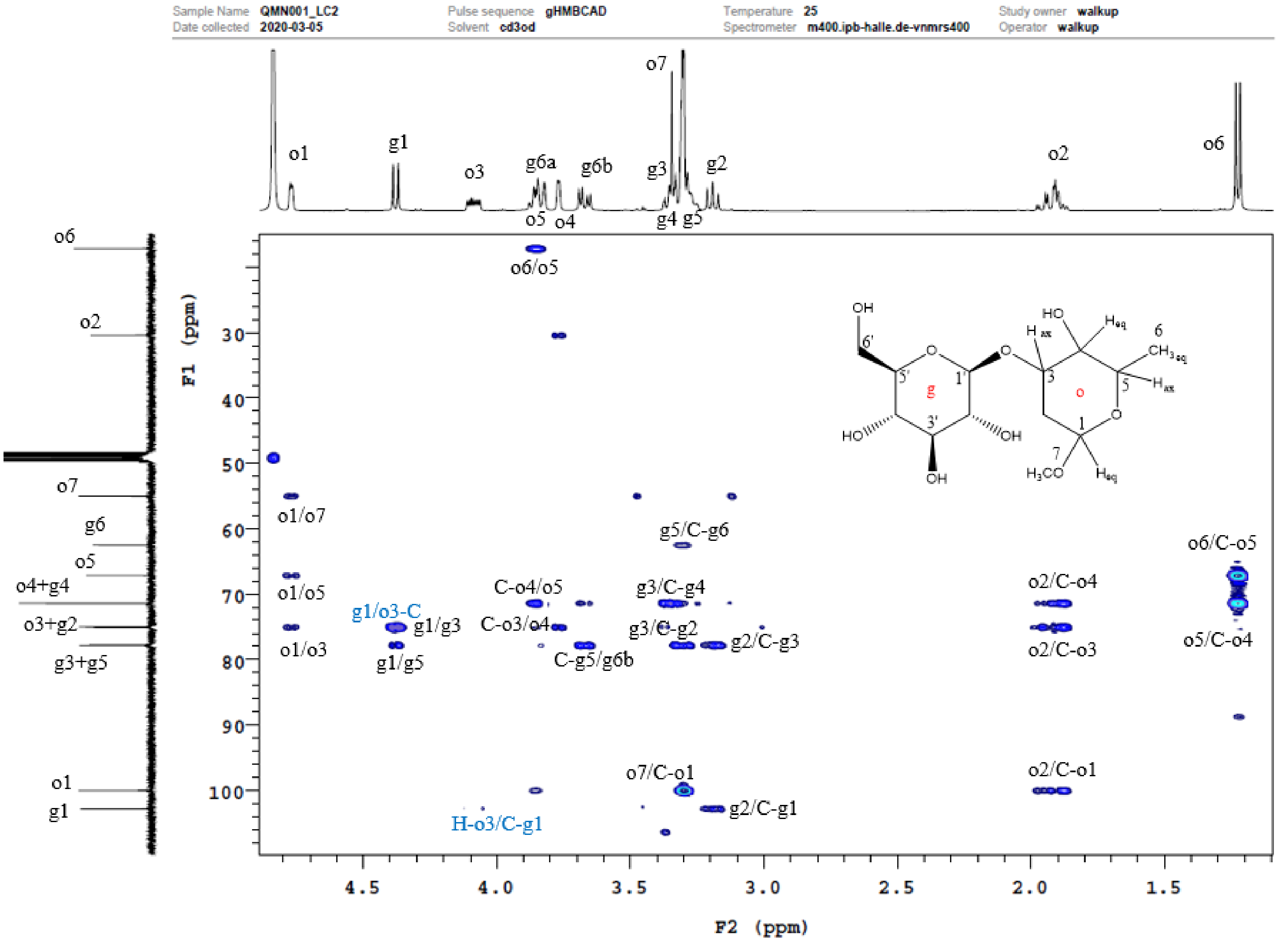

Figure S6. HMBC spectrum of 1-methoxy-3-O-β-glucopyranosyl-α-L-oliose (**1**) in CD_3OD

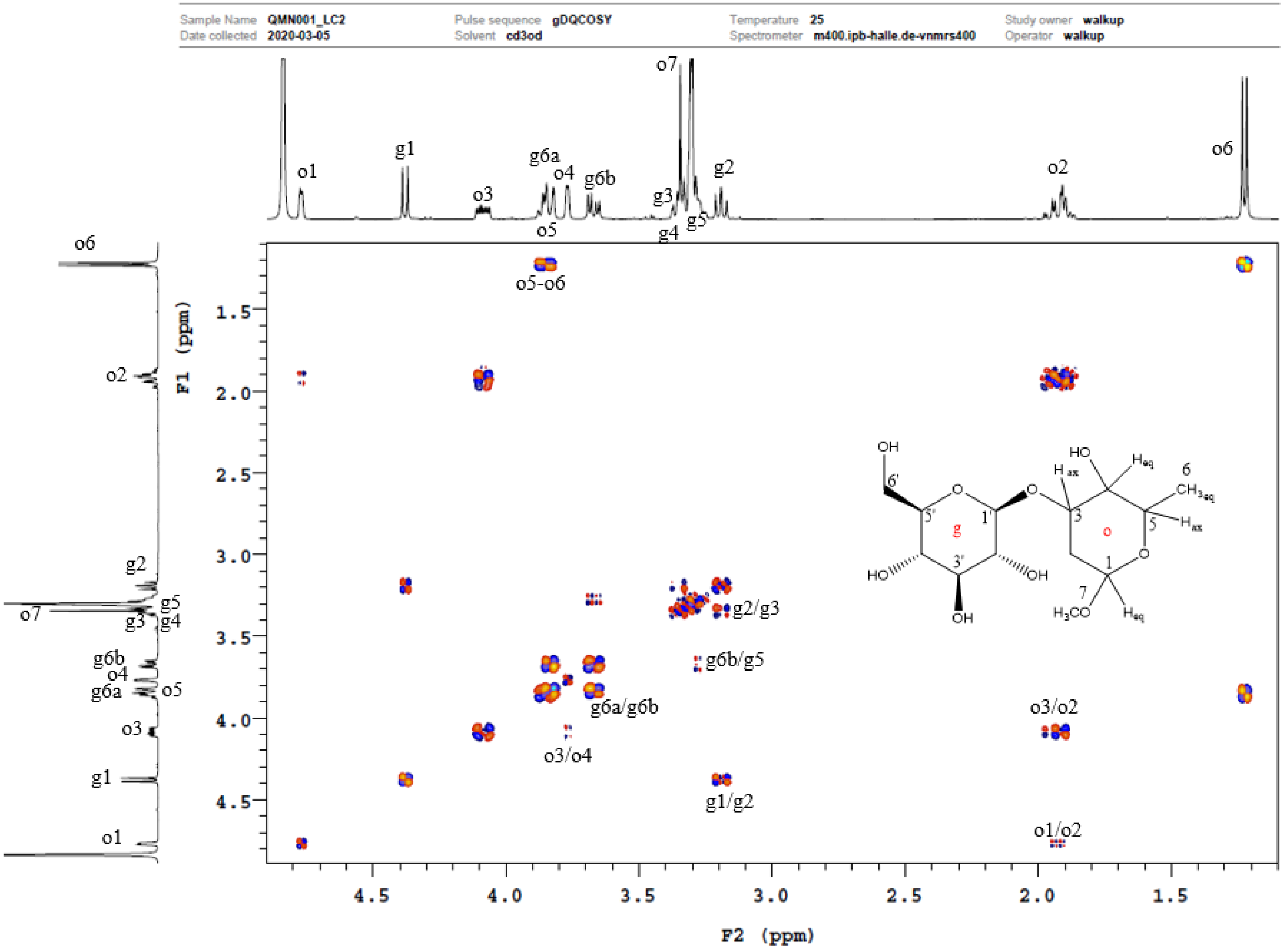

Figure S7. COSY spectrum of 1-methoxy-3-O-β-glucopyranosyl-α-L-oliose (**1**) in CD_3OD

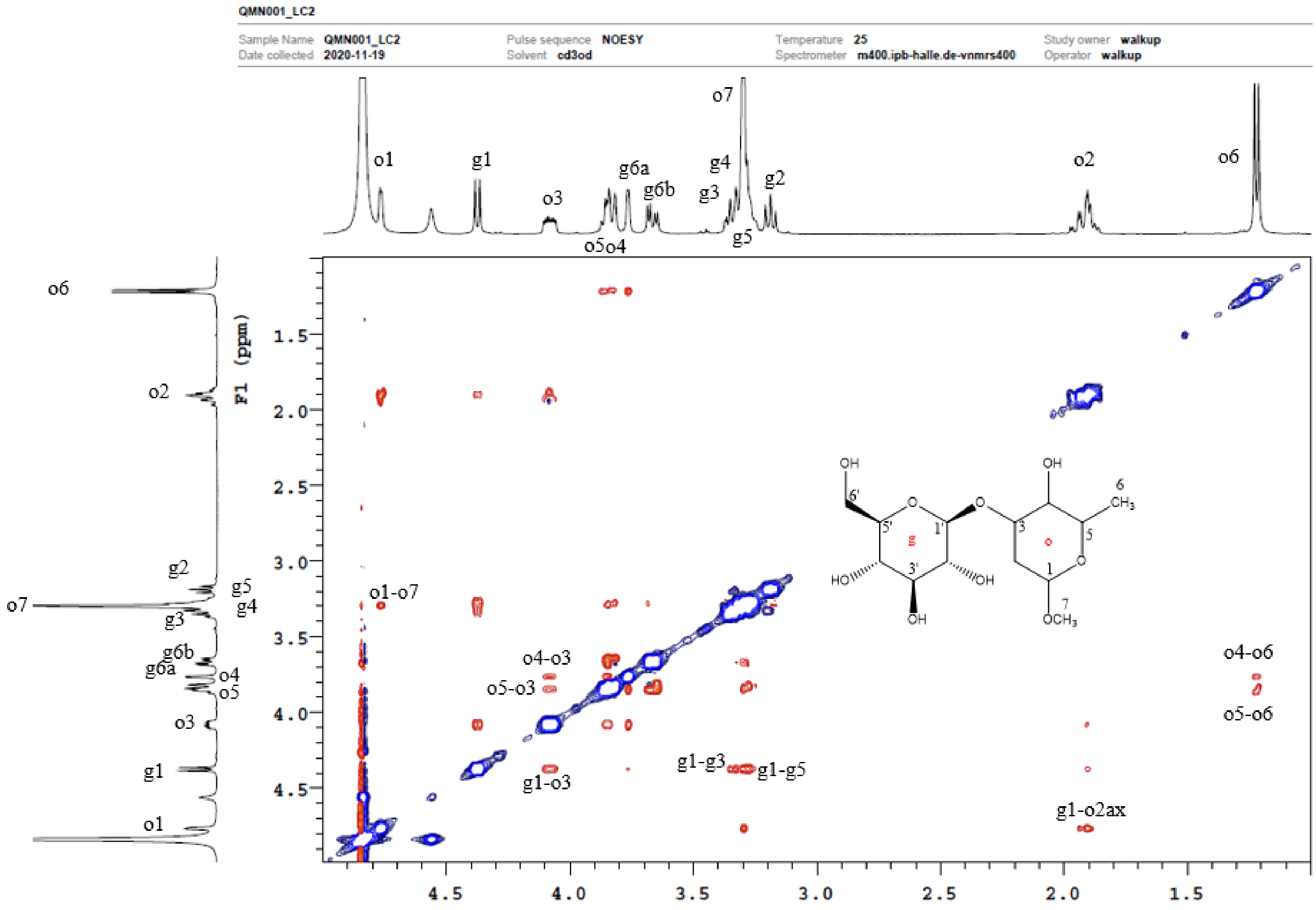

Figure S8. NOESY spectrum of 1-methoxy-3-O-β-glucopyranosyl-α-L-oliose (**1**) in CD_3OD

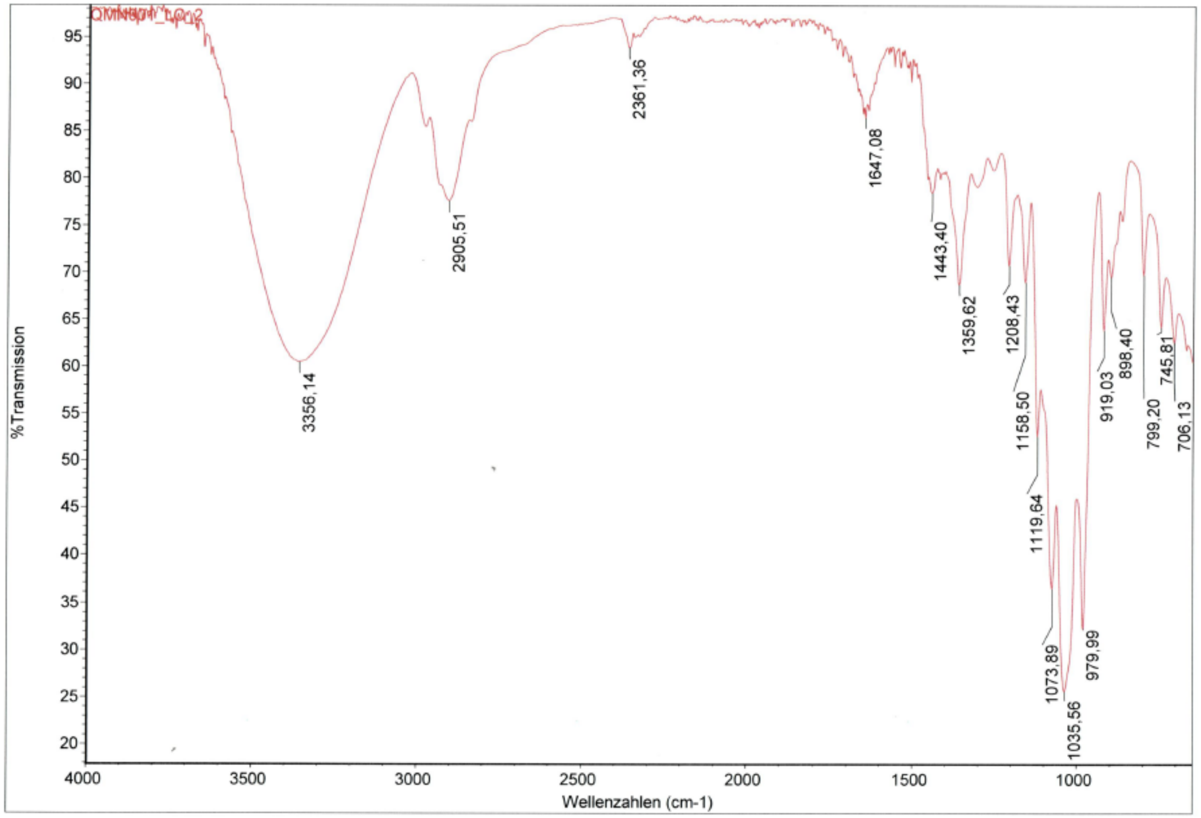

Figure S9. IR spectrum of 1-methoxy-3-O-β-glucopyranosyl-α-L-oliose (**1**) (spectra were measured in ATR mode)

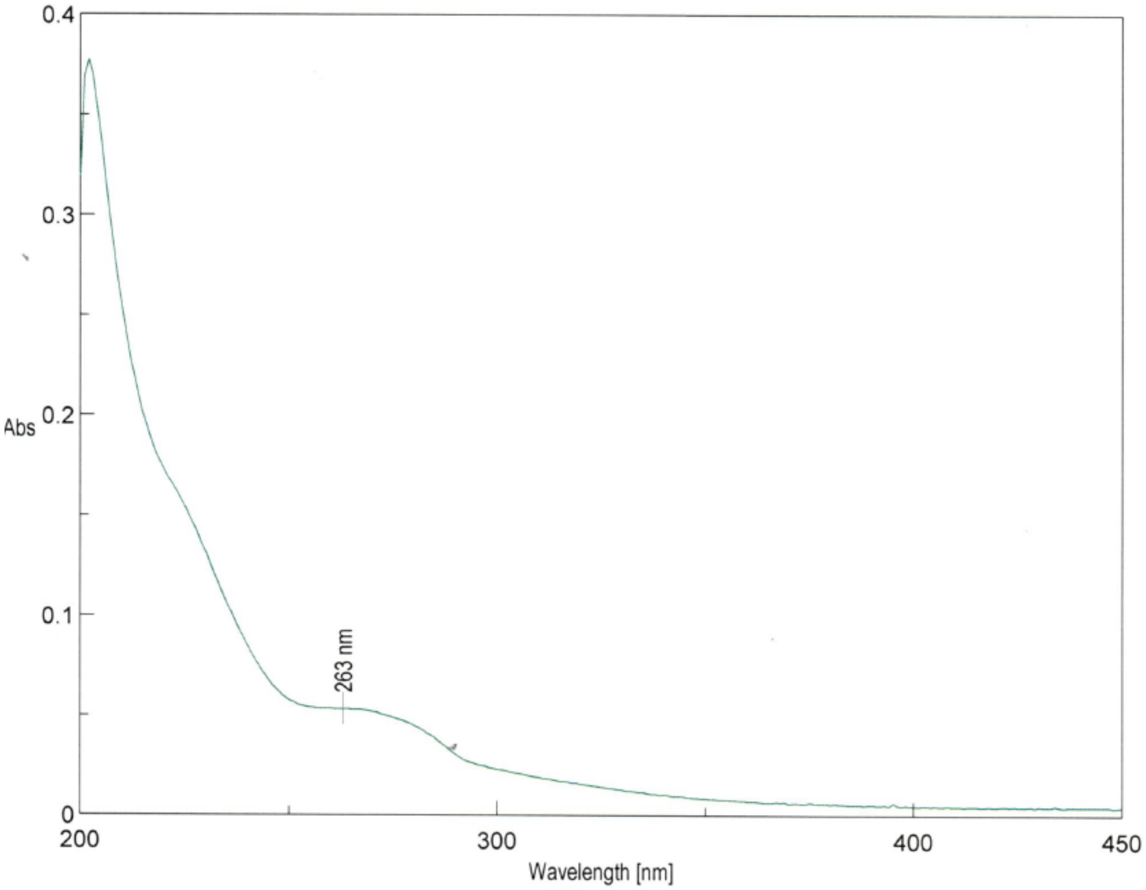

Figure S10. UV spectrum of 1-methoxy-3-O-β-glucopyranosyl-α-L-oliose (**1**) in CH_3OH
